# Controversies in Selecting Nobel Laureates: An Historical Commentary

**DOI:** 10.5041/RMMJ.10479

**Published:** 2022-07-31

**Authors:** Marshall A. Lichtman

**Affiliations:** 1 Professor Emeritus of Medicine and of Biochemistry and Biophysics, James P. Wilmot Cancer Institute, Rochester, NY, USA; 2 Dean Emeritus, School of Medicine and Dentistry, University of Rochester Medical Center, Rochester, NY, USA

**Keywords:** Alfred Nobel, Nobel Foundation, Nobel laureates, Nobel Prizes

## Abstract

There is universal agreement that the Nobel Prizes, given to individuals who have made an extraordinarily notable contribution to humankind in the fields of chemistry, physics, physiology or medicine, literature, and peace, are the most prestigious prizes offered for human achievement. This commentary gives an overview of the basis for Alfred Nobel writing his third will that established the five prizes and includes a discussion of why those five fields were chosen. The commentary includes factors that influenced his choices and contains examples of controversial selections or omissions, especially in the earlier years. A few were errors of omission (e.g. Tolstoy, Tesla, Edison, Best, Gandhi, Franklin), some errors of commission (e.g. Fibiger, Moniz); but, given the complexity of the task, the error rate is small. In some cases, the conclusion that an error had been made is debatable. Such decisions are difficult. Arne Tiselius, a Nobel laureate in chemistry and President of the Nobel Foundation said that one cannot in practice apply the principle that the Nobel Prize should be given to the person who is best; it is impossible to define who is best. Hence, there is only one alternative: to try to find a particularly worthy candidate. This paper includes a brief review of the integration of the Sveriges Riksbank Prize in Economic Sciences in Memory of Alfred Nobel, established in 1968, and added to the original five Nobel Prizes; the prize was first awarded in 1969. A short discussion on the absence of a Nobel Prize in mathematics is provided. Adaptations to the development of “big” science, especially in physics, may require the Nobel Foundation to extend its limit of no more than three awardees for the prize in physics and, perhaps, other scientific disciplines.

## INTRODUCTION

Every year since 1901, except for a few years during World Wars I and II and on a few occasions in the early years when a worthy candidate could not be identified for a specific prize, the Nobel Prizes have been awarded in Stockholm and in Oslo (the Nobel Peace Prize) on December 10, the date Alfred Bernhard Nobel (1833–1896) died. On that date, the four laureates in chemistry, physics, physiology or medicine, and literature receive their gold medal and monetary prize, give their Nobel lecture, and are feted at an elegant banquet hosted by the King and Queen of Sweden in the Stockholm City Hall. Since 1969, the Sveriges Riksbank Prize in Economic Sciences in Memory of Alfred Nobel has been integrated into the selection process and ceremony in Stockholm. The peace laureate is honored at a ceremony in the Oslo City Hall at which members of the Norwegian royal family, the Storting (parliament), and other government offices, and prominent Norwegians and members of the diplomatic corps are among hundreds of guests. The Nobel Peace Prize banquet is held in Oslo’s Grand Hotel, sometimes preceded by a torchlight procession. The events in Stockholm and Oslo go unheralded since the media coverage and the associated hoopla occur at the time of the announcement of the winners, two months earlier, in October, the month of Nobel’s birth. In 2020 and 2021, due to the COVID-19 pandemic, the ceremonies in Sweden were interrupted and the laureates received their medal and check for well over one million dollars in their countries, delivered by a Swedish official. A much scaled-down ceremony with masks was held in Oslo to deliver the Nobel Peace Prize.

The decision by Nobel to have the Norwegian Storting determine the recipient of the Nobel Peace Prize irritated the Swedish King and had him pondering, briefly, a refusal of Sweden as the seat of the Prizes. At the time of Nobel’s death in 1896, the union between the Kingdoms of Norway and Sweden under the Swedish House of Bernadotte was fragile. Norwegians wanted an independent government and full national identity. The King may have thought Nobel’s inclusion of Norway indicated his support for separation. After eight years of political agitation by Norwegians, a peaceful resolution of the dispute occurred, four years after the Nobel awards had commenced. A plebiscite in Norway resoundingly backed dissolution of the union. Following months of tension and concern regarding war between the adjacent countries, King Oscar II of Sweden (1829–1907) deliberated on his options. As a result, he renounced his claim to the Norwegian throne. After having spent nearly one century as the United Kingdoms of Sweden and Norway, the two nations peacefully severed their union. Prince Carl of Denmark (1872–1957) became King of Norway, based on another plebiscite of Norwegians followed by approval by the Storting. Norway became an independent constitutional monarchy in October 1905. Perhaps, the establishment of the Nobel Peace Prize in some way contributed to the King of Sweden rejecting war as a way to retain Norway under Sweden’s royal dominance.

Initially, upon hearing of the stipulations in Nobel’s will, the King of Sweden thought it disloyal that the prizes were not confined to Swedes. Had they been, the awards would have gone unnoticed outside that country. Ultimately, he accepted the directive and it remained open to the man or woman selected regardless of nationality, as Nobel’s will had specified.

A specific Nobel Prize may be shared for no more than two different areas of inquiry and by no more than three persons in any one year. These requirements, invoked by the Nobel Foundation, not in Nobel’s will, understandable as they are, have led to controversies. In some cases, key contributors were omitted either as a result of these arbitrary limits or because sufficiently exhaustive research on the development of a discovery was not done. In some cases, the Nobel lecture has been used by laureates to acknowledge forerunners or colleagues omitted. These oversights have been notable among the Nobel Prizes in Physics, Chemistry, and Physiology or Medicine because of the nature of discovery in those fields, which often is stepwise, building on critical prior observations. Isaac Newton (1643–1727) remarked in his letter to Robert Hooke (1635–1703) in 1675: “If I have seen further, it is by standing on the shoulders of giants,” as symbolic of the nature of scientific advances. Robert King Merton (1910–2003) examined the origin of the metaphor in his monograph, *On the Shoulders of Giants*, published in 1965.

Nevertheless, the Nobel Prizes in Chemistry and Physics generated little controversy as to merit in the early years, although important work inevitably went unrecognized. The two prizes that were less rigorously defined, in peace and in literature, would be subject to considerable second-guessing, and that activity continues. Arne Wilhelm Kaurin Tiselius (1902–1971) served as Vice-President and President of the Nobel Foundation, having previously won the Nobel Prize in Chemistry in 1948. The award recognized him for the invention of a moving boundary electrophoresis device, providing the ability to separate the constituents of protein mixtures. When he was President of the Foundation, Tiselius was asked about the Nobel laureate selection process; he responded that one could not, in practice, apply the principle of awarding the Nobel Prize to the best person since it was impossible to define who was best. In his eyes there was only one alternative, to try to find a particularly worthy candidate. In the earlier years, the Nobel Prize in Physiology or Medicine also had some erroneous selections or controversial omissions made by the Nobel Foundation.

## SOME CONTROVERSIES IN SELECTION OF NOBEL PRIZE LAUREATES

### The Nobel Prize in Physics

In 1912, the choice of laureates in physics was notable for its omissions. One story indicated that the Nobel Foundation was poised to name Nikola Tesla (1856–1943) and Thomas Edison (1847–1931) as joint recipients of the physics prize, a rather heady combination. A dispute between the two, ostensibly, resulted in Tesla indicating he would not share the award with Edison. Presumably, unable to resolve this matter, the Nobel Foundation selected Nils Gustave Dalen (1869–1937), a Swede, who invented a device to allow the gaslight in a lighthouse to automatically turn on at sunset and turn off at sunrise. Few thought this energy-saving device sufficient to justify the prize, especially when compared to the profundity of the discoveries and inventions of Edison and Tesla. One unconfirmed rumor was that the Nobel Foundation chose this path, ostensibly, to stay out of the controversy between Edison and Tesla. Ironically, Tesla received the Edison Medal in 1916, “for meritorious achievements in his early original work in polyphase and high-frequency electric currents.” The American Institute of Electrical Engineers gives the award to recognize a resident of the United States (US) and its dependencies, or of the Dominion of Canada, “for meritorious achievement in electrical science or electrical engineering or the electrical arts.” Neither Tesla nor Edison ever received the Nobel Prize in Physics.

While studying in the Swiss Federal Polytechnic School, Albert Einstein (1879–1955) had recognized the inconsistency between Isaac Newton’s theory of gravity and James Clerk Maxwell’s (1831–1879) theory of light, the two pillars of nineteenth-century physics. However, one of their theories had to be wrong. Eventually, Einstein deduced that the speed of light was a constant, making Newton’s theory unsustainable. In 1905, Einstein published four papers in the *Annalen der Physik*, which revolutionized the discipline of physics. One paper, titled “On the Electrodynamics of Moving Bodies,” contained the mathematical theory of special relativity. Another—”Does the Inertia of a Body Depend upon its Energy Content?”—established that relativity, as defined by Einstein, led to the equation *E*=*mc*^2^, where *E* is energy, *m* is mass, and *c* is the speed of light, 186,000 miles/second. This provided the first mechanism to explain the energy source of the sun and other stars. It also formed the basis for atomic energy and the nuclear age. His work was largely ignored until Max Planck (1858–1947) gave it credence. Planck’s standing was such that Einstein was invited to lecture at international meetings, leading to his rapid rise in academia. He was offered positions at prestigious institutions and, ultimately, at the University of Berlin, where he served as director of the Kaiser Wilhelm Institute for Physics from 1913 to 1933. He famously said: “If my theory of relativity is proven correct, Germany will claim me as a German and France will declare that I am a citizen of the world. Should my theory prove untrue, France will say that I am a German and Germany will declare that I am a Jew.” As the National Socialist (Nazi) Party came to influence, and later power, Einstein’s position and life were threatened and the Nazis recruited scientists to denounce him. *One Hundred Authors Against Einstein* was published in 1931. When asked to comment on this denunciation of his theory by so many scientists, Einstein replied that to reject relativity, the word of 100 scientists was not needed, just one fact. A Nazi organization published a magazine with Einstein’s picture and the caption “Not Yet Hanged” on the cover. The virulence of antisemitism in Germany and Europe and the labeling of his ideas as “Jewish Physics” led him to immigrate to the US, where he joined the Institute for Advanced Studies in Princeton, New Jersey.

Although Einstein’s Nobel Prize in Physics was registered as being awarded in 1921, it was only announced, begrudgingly, in 1922, after no physicist was deemed worthy in 1921. By 1920, he was the world’s most renowned theoretical physicist and scientist. Numerous persons nominated his work on relativity for the physics prize multiple times over several years, all of which were ignored by the Nobel Foundation. His nomination in 1922 (for the 1921 prize) was for discovering the photoelectric effect, which indicated that photons of light have more energy at shorter wavelengths. The Nobel Committee decided to give Einstein the award for that discovery to maintain their respectability, but explicitly not for relativity. Historians have suggested that they could no longer ignore Einstein without the Nobel Foundation being ridiculed and diminished by his omission. The physicist who won the award in 1920 for finding anomalies in a nickel–steel alloy was pleasantly surprised when they overlooked Einstein. This was particularly striking because in 1919 an English astronomer, Arthur Stanley Eddington (1882–1944), had provided evidence for the concept of special relativity when, on an expedition to Principe Island to observe a solar eclipse, he found that light from stars was bent by the gravity of the sun as predicted by Einstein’s theory. Moreover, the Nobel Foundation gave Einstein the 1921 prize simultaneously with the Nobel Prize in Physics given to Niels Henrik David Bohr (1885–1962) in 1922. By announcing Einstein with Bohr, some of the focus was taken away from Einstein. The Foundation procedures indicated that if no awardee was named in a given year, the prize could be rolled over to the next year and presented retroactively. Historians of science indicate that Einstein had made some seven to ten other discoveries in theoretical physics that could have resulted in a Nobel Prize. In each case, derivative works were awarded a Nobel Prize without considering Einstein’s critical role in the discovery. A specific example is the Nobel Prize in Physics for the development of the laser in 1964. Einstein had already provided the theoretical basis for lasers in a 1916 paper on spontaneous light emission from atoms, which included a discussion of stimulated emissions.

The omission of a prize for his theories of relativity was conscious. Einstein’s Nobel Prize citation included the statement “… without taking into account the value that will be accorded your relativity and gravitation theories after these are confirmed in the future.” The Foundation’s implication that they might recognize those achievements in the future was never fulfilled, even after several other astronomers confirmed Einstein’s predictions based on relativity. This omission is inexplicable on scientific grounds. Precedent was established for giving two awards to the same individual when Marie Salomea Skłodowska-Curie (1867–1934) received a Nobel Prize for Physics in 1903 and for chemistry in 1911. Later, John Bardeen (1908–1991) received the Nobel Prize in Physics in 1956 and in 1972, and Frederick Sanger (1918–2013) was selected for the Nobel Prize in Chemistry in 1958 and 1980. Moreover, the Foundation did not hesitate awarding Francis Peyton Rous (1879–1970) a Nobel Prize in Physiology or Medicine in 1966, at age 87 years, for identifying a filterable agent (presumptive virus) as the cause of a tumor in chickens, later designated the *Rous sarcoma virus*, 55 years after his evidence was published in 1911. Thus, neither multiple prizes to the same laureate nor time from the achievement to the award could have been an issue for the Nobel Foundation in the failure to recognize Einstein for the Theory of Relativity, or several other landmark contributions.

Much has been written about the Nobel Foundation’s behavior toward Einstein. Einstein was a pacifist and a Jew. The Foundation seemed to prefer physics discoveries based on experimentation and not theoretical physics, and they also may have had difficulty fathoming Einstein’s theories. However, Planck and Bohr were among the many who nominated Einstein for the Nobel Prize. It was discovered posthumously that Allvar Gullstrand (1862–1930), a Swedish member of the selection committee for the 1921 physics prize, and a 1911 laureate in Physiology or Medicine, had written in his diary: “Einstein must never receive the Nobel Prize, even if the whole world demands it.” He did not explain his view.

Einstein did not go to Stockholm to receive his prize in 1922. He was on a speaking tour in Japan. In addition, a German-Jewish official had been assassinated just before the award ceremony, and the investigation indicated that Einstein was one of those on the assassin’s hit list. In light of the political climate of the time, Einstein concluded that he was safer in Japan than in Stockholm. After World War I, among other reasons for the war, the purported malicious behavior of Jews ranked high. Antisemitism was flourishing, and in Germany and Austria the Jews were blamed for losing World War I and the damages imposed on Germany due to the Treaty of Versailles. This conspiracy theory was compounded by the role prominent Jews such as Leon Trotsky (1879–1940) and others played in the Bolshevik Revolution, the establishment of the Soviet Union, and the short-lived communist governments in Hungary and Bavaria, each led by a Jew. Europe and America’s middle classes used these associations to enhance anti-Semitic conspiracy theories. When Einstein was asked what recognition he most appreciated, it was the inaugural Max Planck Medal for theoretical physics in 1929, presented to him by Planck. He never cited the Nobel Prize.

### The Nobel Prize in Literature

In its inaugural year, 1901, the Nobel Foundation had the opportunity to initiate the literature prize by recognizing one of the greatest and most impactful novelists of the day, namely Leo Tolstoy (Count Lev Nikolayevich Tolstoy) (1828–1910). Two of Tolstoy’s novels, *Anna Karenina* and *War and Peace*, are considered masterpieces of literature. The failure to award Tolstoy the prize in literature was greeted by a remarkable response from some of the great writers and thinkers of the period, who wrote a letter venerating Tolstoy and castigating the Nobel Foundation for this gross oversight. Tolstoy was never honored despite five nominations for the literature prize and three for the peace prize. Joyce, Chekov, Proust, Ibsen, and Twain suffered similar fates.

In 1958, the Russian writer Boris Pasternak (1890–1960) was named a Nobel laureate in literature. He had written books of poetry and several autobiographical pieces. The prize, however, was in reaction to his only novel, *Doctor Zhivago*, first translated from Russian to Italian and, subsequently, to English, making it available in the West. It received praise from the world of high literature. He accepted the prize in October, but was not permitted by the Soviet government to receive the formal award in Stockholm in December. Thirty-one years later, his son, Evgeny Pasternak, received the award in Stockholm in his father’s name. Jean-Paul Sartre (1905–1980) refused the Nobel Prize in Literature in 1964. He felt his contributions should be determined by his written words and not by external factors, such as prizes. However, the Nobel Foundation recognizes and lists its awardees whether or not they accept the prize. Nobel’s desire, explicitly stated in his will, was as follows: “… one part to the person who, in the field of literature, produced the most outstanding work in an idealistic direction …” He considered “loftiness of soul and beauty of form” the most important qualities for the work’s recognition. It is thought that these views, expressed by Nobel, led to some of the choices and omissions made as the Nobel Committee grappled with how Nobel’s comments about great literature should influence their choice of the literature laureate, especially in the early years of the prize.

### The Nobel Peace Prize

Nobel’s final will instructed that the peace prize should be given to individuals or institutions that “have done the most or the best work for fraternity between nations, for the abolition or reduction of standing armies and for the holding and promotion of peace congresses.” Much controversy has surrounded some of the laureates chosen, partly because some of the peace-makers were the leaders of opposing warring parties before an accord.

The 1994 peace prize, shared by Yasser Arafat (1929–2004), Shimon Peres (1923–2016), and Yitzhak Rabin (1922–1995) for the agreement attempting to reconcile the Israelis and Palestinians with a “two-state solution,” is one example. Arafat was chairman of the Palestine Liberation Organization (PLO) from 1969 to 2004 and President of the Palestine National Authority (PNA) from 1994 to 2004. Peres was Prime Minister of Israel from 1984 to 1986 and 1995 to 1996 and President of Israel from 2007 to 2014. Rabin was Prime Minister of Israel from 1974 to 1977 and 1992 to 1995. In 1995, Rabin was assassinated by an Israeli fanatic who opposed the result of the negotiations and the peace accords.

Israel agreed to withdraw from Gaza and the West Bank; the Palestinians would have self-determination in those areas. Arafat had been elected President of the self-proclaimed State of Palestine in 1989. In 1993, he recognized the State of Israel by accepting United Nations Resolution number 242 and signed the agreement with Rabin and Peres in Washington, D.C. The implementation of the details of the agreement was pursued for years, later among other Palestinian groups and key representatives and different Israeli leaders who emerged with different attitudes. There was also the complexity of agreements with individual Arab states such as Jordan and Egypt and attempts at agreements with Syria, for example. The dispute has continued with periodic outbreaks of overt hostilities. The two-state solution, fully accepted by both parties in 1993, for which the peace prize was awarded in 1994, is still an aspiration.

Another example of ambiguity was the award shared by Henry Kissinger (b. 1923) and Le Duc Tho (1911–1990) in 1973 for a cease-fire and an agreement to end the American involvement in the Vietnam War. Kissinger was considered the architect of the destructive bombing of the Ho Chi Minh trail through Laos and Cambodia; however, the order came from President Lyndon Johnson (1908–1973), as required by American law. The capture of Saigon in 1975, two years after the prize was made, effectively ended the war. Le Duc Tho did not accept the award as he accused Kissinger and the US of not adhering to the agreement and because the war continued for several years.

One can argue in both of the above cases that the prizes justifiably recognized progress toward the end of overt hostilities.

Failure to award the Nobel Peace Prize to Mohandas Karamchand Gandhi (Mahatma Gandhi, Gandhiji) (1869–1948), after the peaceful transition of British colonial rule to Indian independence in 1947, to which he made a singular contribution, was a striking omission and was widely criticized. *Mahātmā* in Sanskrit means “great soul,” a designation bestowed on Gandhi. He was nominated for the Nobel Peace Prize five times from 1937 to 1948. Indeed, Gandhi’s lifelong commitment to peaceful resolution of disputes was singular over decades of his adult life, before and after he gave up his career as a barrister. The Norwegian Nobel Committee, the representative of the Norwegian Storting, assigned the task of selecting the person to receive the Nobel Peace Prize by Alfred Nobel’s will, presumably was poised to announce Gandhi as the 1948 recipient of the Nobel Peace Prize, but Gandhi was assassinated before the announcement, although this has not been confirmed by the Nobel Foundation. However, the Nobel Prize is not posthumously awarded unless announced before the individual’s death. In 1948, a peace prize was not awarded, and the Nobel Committee indicated that there was no suitable “living candidate” in that year. This statement could have been an indication that Gandhi’s death interrupted the plan to award him the prize in 1948. When the Dalai Lama was awarded the peace prize in 1989, the chairman of the Nobel selection committee said that this award was “in part a tribute to the memory of Mahatma Gandhi.” Indeed, the Dalai Lama shares several traits with Gandi: he is an ascetic Asian, minimalist in dress and expression, and epitomizes and promotes the concept of peaceful resolution of political and social disagreements. The failure to recognize Gandhi by the Nobel Peace Prize may have been the most egregious error in the administration of that prize.

Articles have been written about the undeserving winners of the Nobel Peace Prize. The suggestion has been made that the Foundation should not strain to name someone every year for this prize. Several winners have said they did not deserve the prize, for example Barack Obama (b. 1961). The Foundation’s decision may have been based on Obama’s efforts to negotiate arms reduction with the Russian Federation, to reach out to the Arab World, and his tendency to favor diplomacy over conflict, especially following the term of George W. Bush (b. 1946) and the wars Bush waged in Iraq and Afghanistan. Nevertheless, Obama was left with Iraq, Afghanistan, and Syria to deal with after George W. Bush’s second term, resulting in a peace prize winner being commander-in-chief of armed forces fighting ongoing conflicts. However, the attitudinal shift of this new American President stood in stark contrast to that of the previous (Bush) administration, and the Nobel Prize selection committee felt that this policy change merited recognition and encouragement.

### The Nobel Prize in Physiology or Medicine

On December 30, 1921, a medical team presented their findings to the American Physiological Society in New Haven, Connecticut regarding a pancreatic factor that held the key to controlling blood sugar in diabetic dogs. The team consisted of Frederick Grant Banting (1891–1941), a surgeon of no prior distinction but with an insightful idea and determination; and Charles Herbert Best (1899–1978), a medical student and research associate who worked with the support of John James Rickard McLeod (1876–1935), a Professor of Physiology at the University of Toronto. McLeod gave Banting the use of his laboratory, his student research assistant Charles Best, several dogs with which to work, and research supplies; then McLeod went on a summer vacation. He was skeptical that Banting’s proposal had merit. In a short time, Banting and Best had isolated a crude fraction from the pancreas of dogs. They demonstrated that it contained a factor that could normalize blood sugar in a dog that had been made diabetic by removing its pancreas. Subsequent to their presentation, a biochemist at the University of Alberta, James Bertram Collip (1892–1965), was recruited by McLeod to assist Banting and Best. They isolated and purified insulin from the pancreas of cattle from slaughterhouses with the goal of using it to treat human insulin-deficient juvenile (type 1) diabetes mellitus. Banting tested the product on himself to measure its safety. On January 23, 1922, at Toronto General Hospital, they administered purified bovine insulin for the first time to Leonard Thompson, a 14-year-old boy with type 1 diabetes: the boy’s elevated blood sugar was lowered. Type 1 diabetes, heretofore treated with a starvation diet, and a fatal disease, could now be managed.

In 1922, Eli Lilly and Company began commercial production of insulin. In 1923, Banting and McLeod were awarded the Nobel Prize in Physiology or Medicine. Best and Collip were not included among the prize recipients. In 1922, international communication among scientists was rudimentary. Hence, this omission, presumably, was due to the nominators for the prize being unaware of their contributions. McLeod was a recognized expert on carbohydrate metabolism. Nevertheless, although McLeod had provided the resources for the research, he had no part in the intellectual enterprise. Banting, the prime mover, felt Charles Best deserved to be recognized by the Nobel Foundation and shared his monetary prize with Best. McLeod likewise shared his prize money with Collip. At the fiftieth anniversary of the isolation and application of insulin, the Nobel Foundation indicated that Best should have been included with Banting and McLeod but that they and their carefully selected nominators worldwide had been unaware of his important role. This confession was a very unusual, perhaps unique, *mea culpa* from the Nobel Foundation. The award to Banting, Best, and Collip would have recognized the most relevant scientists responsible for the achievement. Best eventually succeeded McLeod as Professor of Physiology at the University of Toronto and had a distinguished academic career with many honors bestowed as the co-discoverer of insulin.

The 1926 Nobel Prize in Physiology or Medicine was given to Johannes Andreas Grib Fibiger (1867–1928), a Danish physician and professor of pathological anatomy at the University of Copenhagen, for research concluding that the larva of a worm, he designated *Spiroptera carcinoma*, caused gastric cancer in rats. Soon thereafter, this finding was found to be erroneous. In an era during which cancer was a mysterious and usually lethal disease, the Nobel Prize selection committee thought an animal model would propel the field forward. Unfortunately, the lesions were misidentified as neoplastic but were not. A paper published fifteen years earlier proposing *Shistosoma haematobium* as the cause of bladder cancer, along with a long-standing theory that infection was a precursor to cancer, may have contributed to this erroneous decision. Fibiger’s Nobel lecture provides an elaborate basis for his work. Later research did support the etiologic role of selected specific infectious agents as initiators of cancer of some tissues: bacteria (e.g. *Helicobacter pylori*), viruses (e.g. *Human Papilloma Virus*), and parasites (e.g. *Shistosoma haematobium*). A Nobel Prize was awarded for the discovery of *Helicobacter pylori* in 2005 and for the *Human Papilloma Virus* in 2008.

The refusal to recognize Jonas Salk (1914–1995) in the mid-1950s, notably 1955 or 1956, after the highly successful clinical trial of the polio vaccine he developed in collaboration with the March of Dimes, a remarkable fund-raising initiative, the result of which was reported in the spring of 1955, was inexplicable. On April 12, 1955, Dr Thomas Francis Jr (1900–1969), director of the Poliomyelitis Vaccine Evaluation Center at the University of Michigan, School of Public Health, reported that the Salk polio vaccine was safe and had an efficacy of approximately 90% in preventing paralytic polio. The poliomyelitis vaccine had been tested in a two-year national clinical trial in over 1,800,000 children, an unprecedented trial population size. Jonas Salk did his graduate training at the University of Michigan, School of Public Health, Department of Epidemiology, where Professor Francis was chair of the department. Members of the Nobel Committee to select the awardee in physiology or medicine argued that Salk made only a technical achievement, not worthy of the prize. This willful omission flew in the face of Alfred Nobel’s expressed wishes, articulated in his will, that the prizes should be awarded, as he stated: “to those who, during the preceding year, shall have conferred the greatest benefit on mankind.” The Nobel Foundation, of course, could not adhere in general to the stipulation “in the preceding year.” Nevertheless, Salk’s selection in 1955 or 1956 would have represented the embodiment of Nobel’s intention.

Millions worldwide rejoiced that the expected annual polio epidemics were now preventable, this author’s parents included. Furthermore, three individuals were chosen to share the 1956 prize in physiology and medicine for developing the insertion of dye via cardiac catheterization to enhance study of the coronary circulation and intracardiac pressures. While no doubt a worthy choice, this was also a technical achievement and did not have the immediate and dramatic impact of the polio vaccine.

Salk’s notable contribution to humankind was most likely overlooked due to some committee members’ misjudgment. There was also an unpleasant rivalry between Salk and Albert Bruce Sabin (1906–1993), primarily generated by Sabin, who did not believe a killed viral vaccine was the way to immunize against the polio virus. He believed in an attenuated live viral vaccine. He ultimately developed a live virus vaccine and ran successful trials in Russia, Mexico, and other sites. The Salk vaccine had been proven safe and effective several years before the Sabin vaccine was approved. Neither Salk nor Sabin, who both developed successful polio vaccines, were ever selected for the Nobel Prize. In 1954 John Franklin Enders (1897–1985), Frederick Chapman Robbins (1916–2003), and Thomas Huckle Weller (1915–2008) received the Nobel Prize in Physiology or Medicine for developing a technique to grow poliovirus in the laboratory using tissues other than neural. The three were distinguished virologists, each having advanced the fields of virology and vaccinology. They made important discoveries and contributed to humankind. Their work enabled Salk to grow poliovirus in a manufacturing facility for inactivation and use in his killed-virus vaccine. Enders thought Salk did not appropriately share credit with others who made it possible to develop the vaccine. Apparently, several leading figures in virology and vaccinology disliked Salk, which contributed to the feeling that he was unworthy of the Nobel Prize. Nevertheless, liked or not by some, his development of the vaccine, the enormous clinical trial, and its success, witnessed by its continued use over the last 67 years, demonstrate that Salk’s contribution to humankind was worthy of the prize.

In 1935, a Portuguese neurologist and neurosurgeon, António Caetano de Abreu Freire Egas Moniz (1874–1955), introduced the neurosurgical prefrontal leucotomy procedure (a.k.a. prefrontal lobotomy). This procedure severs the connection between the prefrontal cerebral cortex and the neighboring brain. He used this procedure on patients with schizophrenia, severe depression, panic disorder, and mania and reported positive results. However, prefrontal lobotomy could cause significant side effects, including behavioral and personality deterioration or a vegetative state. Moniz argued the net effects were beneficial. Because of the absence of pharmacological therapy at that time and the urgent need to find treatments for severe mental illnesses, it gained credibility in European centers and the US.

Joseph P. Kennedy Sr (1888–1969) was concerned that his daughter Rosemary (1918–2005) would stain the family’s reputation because of an injury at birth that led to occasional erratic behavior. He was concerned that her social behavior, sometimes promiscuous, would lead to a child out of wedlock and injure the family’s standing. At that time, he had political ambitions for himself and his oldest son, Joseph Jr (1915–1944). Without anyone’s concurrence, he enlisted two neurosurgeons at The George Washington Medical Center to perform a lobotomy on Rosemary in 1941 at 21 years of age. His wife, Rose Kennedy (1914–1969), said she was unaware of the surgery until it was over. Informed consent was not a formalized medical concept, and, presumably, Rosemary had no say in the decision. She went from being an interacting, engaging young woman to a profoundly disabled state, unable to talk or walk. Rosemary was institutionalized for the rest of her life and died in 2005, having outlived six of her eight siblings. These events were thought to have influenced her brother, President John F. Kennedy (1917–1963), and her sister, Eunice Kennedy Shriver (1921–2009). President Kennedy supported and signed the Maternal and Child Health and Mental Retardation Planning Amendment to the Social Security Act. Shriver established the Special Olympics to recognize the achievements of the physically and mentally disabled. Shriver was a tireless force who gave recognition to a population without a voice. Her sister’s disastrous surgery was a terrible price to pay. Still, this episode may have led Rosemary’s powerful and influential siblings to provide needed attention to the cause of a caring and supportive environment for those with intellectual disabilities.

In the fall of 1962, the establishment of the US National Institute of Child Health and Human Development was another significant outcome of Rosemary Kennedy’s disastrous surgery, her brother’s role as President of the US, and her sister’s influence. The US Surgeon General could create new Institutes within the authority of the US Public Health Act. However, the National Institutes of Health (NIH) were established to study specific diseases of the heart, lung, kidney, brain, musculoskeletal system, infectious diseases, and more. Hence, an institute on behalf of children was not welcomed by the NIH leadership, who did not want their mission altered and did not believe children’s health was a national priority. The NIH director was quoted as saying, “… a good grandmother could provide most of the care required by infants and children.”

Eunice Shriver intervened with her brother, the President, and convinced him to make child health a priority of the new administration. President Kennedy indicated in a message to Congress that he planned to establish a National Institute of Child Health and Human Development. The enabling authority of congressional legislation was thought to be needed. Eunice Shriver led an effort to lobby Congress to pass the legislation required and to ensure that the word “child” would be included in the new institute’s name. The consequence of this initiative has been the investigation of the causes, manifestations, and treatment of many very consequential childhood illnesses. It also opened the door for institutes devoted to other epochs of life, such as aging.

Moniz, remarkably, shared the Nobel Prize in Physiology or Medicine in 1949 for his neurosurgical procedure. Later, after the procedure was considered dangerous and unethical, an unsuccessful effort was made to get the Nobel Foundation to rescind the prize to Moniz.

Much has been written about the omission of Rosalind Elise Franklin (1920–1958) in the award to James Dewey Watson (b. 1928), Francis Harry Compton Crick (1916–2004), and Maurice Hugh Frederick Wilkins (1916–2004) for decoding the structure of DNA. Franklin was an innovative crystallographer and an excellent organic and biological chemist. Watson and Crick were not. Before she had a chance to publish the results, Wilkins took Franklin’s crystallographic image #51 from her laboratory and showed it to Watson without her knowledge. This experience led to Watson’s eureka moment when her discovery made it apparent to him that the molecule was a double helix. Among a few others, she also pointed out that the hydrophilic phosphates must point outward, and the hydrophobic bases should point inward. Initially, Watson and Crick had it reversed. Remarkably, Watson and Crick’s one-page paper in Nature published on April 25, 1953 resulted in the prize. They had no experiments, just the knowledge that the molecule was composed of nucleic acid bases, phosphate, and a pentose sugar, deoxyribose. The four bases had been identified as adenine (A), guanine (G), cytosine (C), and thymidine (T). Furthermore, it had already been known that regardless of the DNA source, from salmon roe to human tissue, the molar ratio of CG and AT was always 1 to 1, strongly suggesting that they were paired. Watson and Crick’s concept, based partly on the three constituents, the possible relationship of base pairs, and the crystallographic data, resulted from keen insights and was a momentous achievement. Moreover, Crick was a physicist and had only a few years before turned to biology under the tutelage of Max Perutz. In 1953, when the structure of DNA was published, Watson was 25 years old and had completed his one-year postdoctoral two years previously. Their work answered, arguably, the most important question in biology, but it would be nine years before the Nobel Committee selected Watson and Crick along with Wilkins for the 1962 Nobel Prize in Physiology and Medicine.

Franklin had died from ovarian cancer in 1958 and, thus, was ineligible to receive the Nobel Prize. She and Wilkins could have shared the prize in chemistry before her death, and Watson and Crick the prize in physiology or medicine to meet the requirements of no more than three awardees for any prize in any given year. This decision would have been an unusual workaround; however, elucidation of the structure of DNA is the most significant discovery in molecular biology to date. Its publication represents one of the three most impactful papers in biology, along with Gregor Johann Mendel’s (1822–1884) studies of pea plant inheritance, the foundational elements of what became Mendelian genetics, and Charles Robert Darwin’s (1809–1882) *On the Origin of Species by Natural Selection*. Many think misogyny was a part of the unfair outcome. The Royal Mint in the United Kingdom has instituted a commemorative 50 pence coin issued periodically to highlight their “Innovation in Science” series. The first was dedicated to Stephen William Hawking (1942–2018). On July 25, 2020, the 100th anniversary of Rosalind Franklin’s birth, the Mint dedicated the second coin to her. On one side of the coin is a replica of image 51 showing the double-helical image of DNA. Her name is displayed vertically on the coin, and her first and last name end in “D” and “N” to which, cleverly, they added an “A,” horizontally.

## WHY DID NOBEL CHANGE HIS WILL AND ESTABLISH THE FIVE PRIZES?

In 1888, Parisian newspapers erroneously reported the death of Alfred Nobel, then living in Paris, when it was his brother, Ludvig Nobel (1831–1888), who had died in Cannes from severe atherosclerotic heart disease. The erroneous obituary of Alfred, when it was Ludvig who had died, was headlined “Le marchand de la morte est morte” (“The merchant of death is dead”). It continued by stating that Alfred Nobel became rich by “... finding ways to kill more people faster than ever before.”

Ludvig and their brother Robert Nobel (1829–1896) had built an enormous oil company, Branobel, which eventually produced over half the kerosene sold to the Russian Empire. The Nobel family business started with developing explosives for canal building, notably at Suez. The business morphed into the manufacture of armament (e.g. cannons), land and sea mines, and other munitions at the behest of Tsar Nicholas I (1796–1855) to support Russia’s involvement in the Crimean War in the mid-1850s. Alfred Nobel had many inventions to his credit. But the most notable was dynamite, a mix of nitroglycerine and diatomaceous earth. Nitroglycerine, a blasting oil, had been used in the past, resulting in numerous tragic accidents. The addition of diatomaceous earth provided a stable, safer product that retained its explosive power upon detonation. Alfred Nobel’s worldwide patents for dynamite contributed to his enormous fortune, bolstered by his share of his brothers’ oil empire.

Subsequently, Alfred Nobel invented blasting gelatin: gelatinized glycerin with a small fraction of nitrocellulose. It was stable, as powerful as dynamite, and could explode under water, giving it a broader utility than dynamite. Blasting gelatin doubled the rate at which tunneling through a mountain could be accomplished when compared to dynamite.

Alfred Nobel wrote three wills over the years. According to his first will, Alfred Nobel’s earlier intentions involved leaving his fortune to the children of siblings, loyal servants, several friends, employees and colleagues, and a former paramour. The last-mentioned, a younger woman, Sofie Hess, was a sales girl in a flower shop, with whom he sustained a long and somewhat erratic relationship. She was 20 years of age and he 43 years when they met in the shop. Their twenty-year relationship was deep, emotional, but apparently platonic, as evidenced by archives of numerous letters they wrote to each other, especially his letters to her.

A few years before his death, Nobel wrote a second will in 1893, and then executed a third will in November 1895. This final will established the five Nobel Prizes, leaving smaller, but substantial amounts of money to several individuals, including Sofie Hess, who had married and had a child. The erroneous obituary of Alfred Nobel in the French newspaper, with its unflattering description of him as a supplier of weapons of war and death, is thought to have influenced this third will. By changing his bequests to mostly family, colleagues, and friends to a different cause, he would be less likely to be viewed as an arms merchant. Alfred Nobel’s decision was notable because in life he had derided awards when offered to him. This third and final will was challenged at several levels: the fraction of his wealth being distributed to beneficiaries, the taxes to be imposed by France, defining of his place of residence (Alfred Nobel had not lived in Sweden since the age of nine), the sale of Nobel’s physical holdings, the placement of the peace prize in Norway, the international character of the prize recipients, and more. The most contentious issues were resolved by skilled executors and a nephew, Emmanuel, Ludvig Nobel’s son, who argued persuasively that his uncle’s wishes should be respected precisely. The newly established Nobel Foundation also had to devise the methods to assign each prize to an appropriate Swedish academic institution to provide the expertise for the laureate selection process. It would take five years for the will’s intent to be executed due to its poor preparation by Nobel, and its content and proposals, which surprised the executors. Hence, the Nobel Prize was only initiated in 1901, five years after his death.

## WHY THE FIVE DISCIPLINES FOR PRIZES: CHEMISTRY, PHYSICS, PHYSIOLOGY OR MEDICINE, LITERATURE, PEACE AMONG NATIONS?

### Chemistry, Physics, and Physiology or Medicine as Foci for Three Prizes

The choice of the disciplines among which to select Nobel laureates, not surprisingly, relates closely to Nobel’s interests. As a teenager, Nobel was intrigued by chemistry, being tutored in the discipline in St Petersburg, where his family had moved to build their armaments industry on behalf of the Tsar and Russia’s military needs and ambitions. Chemistry, notably, and physics were his lifelong scientific interests and the basis for his and his family’s achievements and contributions to explosives developed and used in construction. The explosives were in high demand for a range of massive projects, including tunneling railways or roadways through mountains and digging pathways for canals. Alfred Nobel held 355 patents, most related to industrial chemistry; only Thomas Edison had more.

When considering the effort to recognize accomplishment in providing a benefit to humankind, the prize in physiology and medicine is understandable. Nobel had suffered from cardiovascular disease and had angina pectoris; he eventually died of a stroke. His angina was treated with nitroglycerin, which was the essential explosive in his invention of dynamite. Nitroglycerin was invented by an Italian, but its instability made it very hazardous to use. Nobel’s younger brother and several others were killed in an accidental explosion while handling nitroglycerin in a Nobel laboratory in Sweden. After much research, Nobel found that nitroglycerin could be stabilized by adding kieselguhr (diatomaceous earth), making it safer to handle. He named the mixture “dynamite” after the Greek word for power, “dunamis.”

Nitroglycerin’s medicinal property was discovered in a Nobel plant in Sweden that manufactured the explosive. Workers who inhaled the plant fumes complained of headaches that disappeared over the weekend. Interestingly, workers with angina noted relief during the week and a return of symptoms on the weekend. This observation eventually led to development of tiny nitroglycerin tablets flavored with sugar. Over a century later, nitric oxide, the key ingredient, was found to be a vasodilator. A Nobel Prize in Physiology or Medicine was awarded in 1998 to three scientists for the discovery of the role of nitric oxide in vascular tone regulation. The omission of Salvador Moncada (b. 1944) was so egregious that one of the three awardees, Robert Furchgott (1916–2009), commented: “I feel that the Nobel Prize committee could have made an exception this year and chosen a fourth person, Salvador Moncada (to share the prize).” Indeed, Moncada was the first to demonstrate that nitric oxide is a biological mediator in the cardiovascular system, which was published in the prestigious *Nature* in 1987.

### Literature as a Focus of One Prize

The literature prize reflected Nobel’s deep personal interest in the written word. Nobel had little formal education, although he received intensive tutoring as a teen in Russia. Nevertheless, he had a lifelong devotion to literature. He had considered not pursuing a career in the family business and devoting himself to a career in writing. His father convinced him to remain involved in their development of explosives and armament. He was fluent in Swedish, Russian, English, French, and German. When Nobel left Paris to move to San Remo, Italy, where he died, there were approximately 1500 volumes in his library, many in their original language. Most of his collection was fiction. It included the works of nineteenth-century authors, great Russian authors, highly regarded Nordic works, the classics—including the works of Shakespeare—and a variety of disciplines: poetry, religion, philosophy, history, and science. He also had a large collection of personal letters. Nobel had written several poems, drafted the essence of several novels, and completed the script of at least one play entitled “Nemesis.” Several critics thought his particular skill was in poetry. His devotion to language and written expression was lifelong. During the period he lived and worked in Paris, he interacted with the literati, visited literary salons, interacted with contemporary writers, and had a special personal relationship with Victor Hugo.

### Peace among Nations as a Focus for one Prize

The choice of a prize for someone contributing to peace among the nations of Europe almost certainly resulted from his deep and long-standing association and friendship with Countess Bertha Sofia Felitas Kinsky von Chinic und Tettau (1843–1914), who subsequently became the Baroness von Suttner. Countess Kinsky was born in Prague to a prominent family, a field marshal’s daughter and the maternal granddaughter of a cavalry captain. She was raised by her mother and a guardian, the latter a member of the Austrian court, in an aristocratic society with strong military traditions. As a girl and young adult, the Countess studied languages and music, read assiduously, and traveled widely. Not being the first son of her paternal grandfather, the Countess’s father did not inherit any of the family wealth. Her mother, widowed, was in financial difficulties, and Bertha, despite her aristocratic status, had to find work to relieve her mother’s financial burden. When 30 years old, she was hired to be the companion and teacher of Baron von Suttner’s four daughters. She developed a romantic relationship with their brother, but the von Suttner family disapproved of their relationship. At age 33, Bertha, decided to leave and responded to an advertisement in an Austrian newspaper in which Nobel was seeking a personal secretary and household manager. She traveled to Paris, was interviewed, and obtained the position. Her work for Nobel was short-lived as she eloped with the von Suttners’ son. After living in the Caucasus region for nearly a decade, they were brought home to the von Suttner’s Austrian castle and integrated into the family. With her father-in-law’s death, her husband became the Baron, and she the Baroness. Bertha had an exceptional interest in world affairs, notably European politics. She became involved with the International Arbitration and Peace Organization, maintained her focus on European affairs, and she particularly desired to encourage harmony among the often adversarial states of Europe. She published two influential books on the topic. The first was *Die Waffen Nieder* published in 1889, which later was published in English with the title translated to *Lay Down Your Arms*, which made her notable in the field and led to her participation in the Hague Peace Conference in 1899.

Over the years, Bertha corresponded with Nobel on the need to foster peace among the nations of Europe. Undoubtedly, this relationship contributed significantly to his decision to provide a prize for the person who best fostered peace among the nations of the world and encouraged the reduction in standing armies and armaments. Bertha had grown up in a family steeped in military tradition, and Nobel’s fortune had come, in part, from a large armaments business to which his ingenuity made a substantial contribution. As an inventor, he was motivated in part by the technical challenge of inventing advanced weaponry. He cynically thought that powerful weapons might prevent war. Nobel was deeply skeptical that it was realistic to expect nations to disarm, or that peace congresses or courts of arbitration would lead them to eschew war. He stated: “On the day that two armies will be able to annihilate each other in one second, all civilized nations will recoil from war in horror and disband their forces” and “I would like to invent a substance or machine so frightfully effective and devastating that it would forever make war altogether impossible.” The nuclear age satisfied Nobel’s desire for the ultimate weapon, but not his prediction about its ability to serve as a means to eliminate war and settle disputes among nations. Nobel underestimated human depravity, which persists to this day. Witness Putin’s use of Russia’s military weaponry to ravage Ukraine, its men, women, and children, its homes and workplaces, now—in 2022—nearly 150 years after Nobel’s views on ultimate weaponry and peace.

Nevertheless, despite his skepticism about peace initiatives, Nobel supported Bertha von Suttner’s plans and efforts. She became a leading proponent of the need to move away from settling disputes by warfare. Her ardent correspondence with Nobel on the importance of this effort was considered seminal in establishing this prize. It is also possible that Nobel’s and his family’s contribution to the arms industry played a role, consciously or subconsciously, in establishing the peace prize and acted as a form of redemption. This reaction was not unexpected after he was described as a “merchant of death.” Indeed, a few years after Ludvig Nobel’s death and the erroneous obituary describing Alfred unfavorably, he wrote to Bertha and indicated that he was considering changing his first will to include a bequest to support a prize for encouraging peace among the nations of Europe. He proposed giving it every five years for a total of six prizes. He reasoned that if peace could not be assured among the nations of Europe in 30 years, it would never happen. She thought it could be achieved sooner and indicated that money, not prizes, would propel the initiative forward.

Nobel prepared a second will, which was executed in March 1893. Twenty percent went to twenty-two individuals. Sixteen percent went to several institutions, among which was the Austrian Society of the Friends of Peace, which was founded by Bertha von Suttner. Included in this will was a fund for medical research given to the Royal Caroline Institute (Karolinska Institutet) in Stockholm, stipulating that the interest of which would be “awarded as a prize for the most important pioneering discovery or invention in the field of physiology and the medical arts” every third year. The remaining sixty-four percent was assigned to the Academy of Sciences in Stockholm for pioneering discoveries in the field of knowledge and progress other than physiology or medical arts, for which he had already provided. There was no specific mention of a prize for literature. The specific stipulation for a peace prize in the second will confirms Bertha von Suttner’s impact on his thinking about his bequests. One can see the beginnings of a formulation about prizes and the areas to be honored, but the full realization of the prizes, the specific areas of achievement, and the institutions to make the awards awaited his third and final will in November 1895.

Both the Baron and Baroness von Suttner were committed to human rights. He was an ardent opponent of the virulent antisemitism that characterized European society, and she was an energetic proponent of women’s rights. She was awarded the Nobel Peace Prize in 1905 for her work, the second woman to win a Nobel Prize and the first woman to win the peace prize.

### The Sveriges Riksbank Prize in Economic Sciences in Memory of Alfred Nobel

The Sveriges Riksbank Prize in Economic Sciences in Memory of Alfred Nobel was established in 1968 by an endowment from Sweden’s central bank, Sveriges Riksbank, to commemorate the bank’s 300th anniversary. After initial consternation regarding the intrusion into Nobel’s plans for his five prizes, it has come to be accepted as a sixth “Nobel Prize.” Administered by the Nobel Foundation, the laureates are selected by the Royal Swedish Academy of Sciences. Thus, the Nobel Memorial Prize in Economic Sciences winners are chosen similarly and are announced along with the five Nobel Prize recipients. The lecture by the awardee and the receipt of the prize occur at the Nobel Prize award ceremony in Stockholm. It is informally referred to as the “Nobel Prize in Economics.” The first prize in economics was awarded in 1969.

### The Absence of a Nobel Prize in the Field of Mathematics

Much has been written about the absence of a Nobel Prize in the discipline of mathematics. It may have been difficult to require that a prize in mathematics be closely tied to a benefit to humanity in the preceding year, the essential rationale Nobel provided for the five prizes he established. Nevertheless, several mathematicians have won Nobel Prizes for their work in the fields of physics and economics.

However, the Abel Prize was established to give the mathematicians a prize equivalent to the Nobel Prize. A similar award was first proposed in 1902 by King Oscar II of the United Kingdoms of Sweden and Norway, but never executed due to the political distractions in the years leading up to the 1905 dissolution of the union between the two countries. The proposal for a prize in mathematics was revisited in 2000. In 2001 the Norwegian government provided the equivalent of approximately twenty million dollars to create a new award, the Abel Prize in mathematics. The prize is named after Niels Henrik Abel (1802–1829), a Norwegian mathematician whose work in algebra had lasting impact, despite his death at the age of 26. Abel’s name is associated with commutative groups, now commonly known as “abelian groups.”

The Abel Prize is awarded annually and affords the field of mathematics a prize at the highest level. An independent committee of international mathematicians selects the laureates. Thus, mathematicians have an award ostensibly equivalent to the Nobel Prize. However, the Abel Prize has not received the same lay interest and media attention due to the long-standing dominant position and name recognition of the Nobel Prizes, and the intellectual distance between popular and higher mathematics. Thus, its luster has not yet reached that of a Nobel Prize, except, perhaps, among mathematicians and their families.

## CONCLUDING THOUGHTS

Nobel’s third will was handwritten and did not have the benefit of legal advice. Nevertheless, it delineated the five prizes and his intention in their regard, and has proven to be one of the most influential personal documents ever written. The Nobel Prize has been, and remains, the most prestigious symbol recognizing extraordinary human accomplishments in (now) six fields. In many cases, the prize has met Nobel’s primary objective: recognizing a benefit to humankind. Nevertheless, it is difficult, indeed impossible, to avoid controversy when making such rarefied decisions. Indeed, in 1855, the French poet and novelist Arsène Houssaye (1815–1896) coined the term “the forty-first seat” for individuals of great accomplishment who were not elected to the Académie Française, which limits its membership to forty individuals. Notable omissions include René Descartes (1596–1650), Jean-Jacques Rousseau (1712–1778), Émile Zola (1840–1902), Jean-Paul Charles Aymand Sartre (1905–1980), and other deserving scholars of accomplishment.

With the advance of science, choices in the objective disciplines of chemistry, physics, and physiology or medicine have more closely met Nobel’s objective of reflecting a benefit to humankind. However, when the Nobel Foundation recognizes fundamental findings, it may take some time for that finding to prevent, diagnose, or treat disease. The work for which the chemistry and physics prizes are awarded, which provides the basis for later applied advances, often results later on in important practical outcomes. Certainly, the first physics award in 1901 to Wilhelm Conrad Röentgen (1845–1923) for his discovery of an unknown type of ray he designated X-ray, “X” for an unknown form, is a quintessential example of such an outcome. Thus, the physics and chemistry prizes sometimes lead to profound medical diagnosis or therapy advances. Choices in the fields of literature and peace, requiring more subjective decisions, have usually been worthy, meeting Arne Tiselius’s expectations.

Changes in the nature of science may require the Nobel Foundation to consider how to recognize mega-projects in physics, such as the Events Horizon Telescope project that connected eight radio telescopes around the world together to form a single earth-sized virtual telescope. In May 2022, the telescope acquired the first direct image of a supermassive black hole, 12 million miles in diameter, in the Milky Way Galaxy. The existence of black holes was predicted in the early 1900s by Einstein’s Theory of General Relativity. Like the peace prize, awards to groups larger than three persons may become appropriate. The advances in fields such as computer sciences may require the Nobel Foundation to consider whether the five original prizes may need an addition, as was done, after some consternation, with the discipline of economics. Alfred Nobel would likely agree that his Foundation should adapt to the changes that have occurred in the 125 years since he wrote his third will.

## Abbreviations

NIH: National Institutes of Health
US: United States.
